# Inhibition of P-Glycoprotein by HIV Protease Inhibitors Increases Intracellular Accumulation of Berberine in Murine and Human Macrophages

**DOI:** 10.1371/journal.pone.0054349

**Published:** 2013-01-23

**Authors:** Weibin Zha, Guangji Wang, Weiren Xu, Xuyuan Liu, Yun Wang, Beth S. Zha, Jian Shi, Qijin Zhao, Phillip M. Gerk, Elaine Studer, Phillip B. Hylemon, William M. Pandak, Huiping Zhou

**Affiliations:** 1 Department of Microbiology and Immunology, Virginia Commonwealth University, Richmond, Virginia, United States of America; 2 Key Laboratory of Drug Metabolism and Pharmacokinetics, China Pharmaceutical University, Nanjing, P.R. China; 3 Tianjin Key Laboratory of Molecular Design and Drug Discovery, Tianjin Institute of Pharmaceutical Research, Tianjin, P.R. China; 4 Basic Medical College, Tianjin Medical University, Tianjin, P.R. China; 5 Department of Pharmaceutics, Virginia Commonwealth University, Richmond, Virginia, United States of America; 6 Department of Internal Medicine/Gastroenterology and McGuire Veterans Affairs Medical Center, Richmond, Virginia, United States of America; 7 School of Pharmacy, Wenzhou Medical College, Wenzhou, P.R. China; Institute of Human Virology, United States of America

## Abstract

**Background:**

HIV protease inhibitor (PI)-induced inflammatory response in macrophages is a major risk factor for cardiovascular diseases. We have previously reported that berberine (BBR), a traditional herbal medicine, prevents HIV PI-induced inflammatory response through inhibiting endoplasmic reticulum (ER) stress in macrophages. We also found that HIV PIs significantly increased the intracellular concentrations of BBR in macrophages. However, the underlying mechanisms of HIV PI-induced BBR accumulation are unknown. This study examined the role of P-glycoprotein (P-gp) in HIV PI-mediated accumulation of BBR in macrophages.

**Methodology and Principal Findings:**

Cultured mouse RAW264.7 macrophages, human THP-1-derived macrophages, Wild type MDCK (MDCK/WT) and human P-gp transfected (MDCK/P-gp) cells were used in this study. The intracellular concentration of BBR was determined by HPLC. The activity of P-gp was assessed by measuring digoxin and rhodamine 123 (Rh123) efflux. The interaction between P-gp and BBR or HIV PIs was predicated by Glide docking using Schrodinger program. The results indicate that P-gp contributed to the efflux of BBR in macrophages. HIV PIs significantly increased BBR concentrations in macrophages; however, BBR did not alter cellular HIV PI concentrations. Although HIV PIs did not affect P-gp expression, P-gp transport activities were significantly inhibited in HIV PI-treated macrophages. Furthermore, the molecular docking study suggests that both HIV PIs and BBR fit the binding pocket of P-gp, and HIV PIs may compete with BBR to bind P-gp.

**Conclusion and Significance:**

HIV PIs increase the concentration of BBR by modulating the transport activity of P-gp in macrophages. Understanding the cellular mechanisms of potential drug-drug interactions is critical prior to applying successful combinational therapy in the clinic.

## Introduction

Human immunodeficiency virus (HIV) protease inhibitors (PIs) are the major components of highly active anti-retroviral therapy (HAART) and have been successfully used to control disease progression in HIV-1 patients. However, the decline in morbidity and mortality has been clouded by the emergence of a number of metabolic derangements [1]. The prevalence of dyslipidemia in patients receiving HIV PIs is more than 50%, which significantly increases the risk of cardiovascular disease (CVD) [2], [3], [4], [5]. Although cellular/molecular mechanisms underlying HIV PI-induced CVD remain to be fully elucidated, sufficient evidence suggests that lipid accumulation, inflammation, and activation of endoplasmic reticulum (ER) stress are all involved in HIV PI-induced cardiovascular complications and metabolic syndromes[3], [4], [5], [6], [7].

Berberine (BBR) is an alkaloid isolated from medicinal plants such as *Rhizoma coptidis* and *Phellodendron amurense*. Although its traditional use mainly focused in various infectious disorders for a long time [8], the lipid-lowering, anti-diabetic and anti-inflammatory activities were shown in many studies during past decades [9], [10], [11]. Several mechanisms including modulation of AMP-dependent protein kinase (AMPK) activity and regulation of tyrosine kinase, Akt and NF-κB signaling are identified to be associated with the beneficial effects of berberine on improvement of obesity-associated lipid dysregulation and inhibition of vascular and intestinal inflammation [12], [13], [14], [15]. Our previous study also indicated that inhibition of ER stress by BBR represents a key mechanism by which this molecule prevents the HIV PI-induced inflammatory response [16]. Therefore, BBR is a promising complementary agent which may be used with HIV PIs for the treatment of HIV infection.

P-glycoprotein (P-gp) is the most widely investigated member of ATP binding cassette (ABC) membrane efflux transporters and has been identified as a major transporter responsible for the efflux of BBR [17], [18]. Similarly, most HIV PIs also have been described as P-gp substrates at both the intestinal barrier and the blood-brain barrier (BBB) [19], [20], [21]. It also has been reported that HIV PIs act as both inhibitors [22] and occasionally inducers of P-gp [23], [24], [25]. Therefore, HIV PIs may alter the pharmacokinetics of P-gp substrates drugs on multiple levels [26], [27].

Macrophages play a pivotal role in the initiation and progression of atherosclerotic lesions. Our previous study demonstrated that HIV PIs accumulate in macrophages and promote foam cell formation, which is the core component of the atherosclerotic plaque. Macrophages represent an important *in vitro* model to screen potential complementary and alternative medicines (CAMs) which may counteract HIV PI-induced cardiovascular complications. Factors that affect accumulation of these drugs into macrophages are therefore important to consider. Concurrently, the expression of drug transporters deserves attention. Recent studies have shown that P-gp is expressed in both human and mouse macrophages [28], [29] and it is likely to influence accumulation of BBR and HIV PIs in macrophages. However, the role of P-gp in the interaction between BBR and HIV PIs has not been elucidated. In mouse J774A.1 macrophages, we already observed a significant enhancement of BBR intracellular accumulation induced by lopinavir (LOPV) [30]. Therefore, our goal was to further explore the potential role of P-gp in HIV PIs-induced increase of BBR accumulation in macrophages. Functional expression of P-gp and a possible inhibitory mechanism was also probed. The results presented herein indicate that P-gp is involved in BBR efflux in macrophages. In addition, HIV PIs increase BBR uptake by inhibiting the activity of P-gp in macrophages. This study provided new important information for future application of BBR in treatment of HIV PI-associated complications in the clinic.

## Materials and Methods

### Materials

Amprenavir (AMPV), ritonavir (RITV), and LOPV were obtained from NIH AIDS Research & Reference Reagent Program. BBR, verapamil, haloperidol, MK571, bromosulfalein, rhodamine 123 (Rh123), digoxin, and general reagents for High Performance Liquid Chromatography (HPLC) were purchased from Sigma (St. Louis, MO, USA). Cell culture medium and supplement components were from Invitrogen (Carlsbad, CA, USA).

### Cell Culture and Treatment

RAW 264.7 mouse macrophages (ATCC, Rockville MD, USA) was cultured in DMEM medium containing 10% heat-inactivated fetal bovine serum (FBS), 100 U/mL penicillin and 100 µg/mL streptomycin at 37°C with 5% CO_2_. THP-1 human monocytes (ATCC, Rockville MD, USA) were maintained in RPMI Medium 1640 supplemented with 10% FBS, 100 U/ml penicillin, and 100 µg/ml streptomycin at 37°C with 5% CO_2_. THP-1 monocytes were treated with PMA (100 ng/ml) for 5 days to facilitate differentiation into macrophages. Wild-type and human P-gp-transfected MDCK cells were kindly provided by Dr. Hongjian Zhang, PharmaResources Co., Ltd., Shanghai, China. MDCK cells were cultured in DMEM supplemented with 10% FBS, penicillin (100 U/mL) and streptomycin (100 µg/mL). HIV PIs, BBR, P-gp selective inhibitors and substrates were dissolved in dimethyl sulfoxide (DMSO) and directly added into the culture medium and incubated for different time periods.

### Measurement of P-gp activities in macrophages

To evaluate the effect of HIV PIs on P-gp activities in macrophages, RAW264.7 cells were treated with HIV PIs (5, 15, and 25 µM) and Rh123 (5 µM) or digoxin (1 µM) for 4 h. After washing with ice-cold PBS for three times, cells were harvested in 500 µL of 1% (v/v) Triton X-100 in PBS. A 5 µL aliquot was used for measurement of protein concentration and the remaining cell lysate was centrifuged at 14,000 rpm for 5 min at 4°C. The amounts of Rh123 was determined using 96-well fluorescence plate reader with excitation/emission wavelengths at 485/530 nm. Digoxin was determined by Liquid chromatography–mass spectrometry (LC/MS). The total amounts of intracellular Rh123 and digoxin were normalized to total protein concentration.

### Quantification of BBR using High Performance Liquid Chromatography (HPLC) Assay

An Agilent 1200 Series HPLC system and a Beckman C18 reverse phase column (5 µm, 4.6 mm×25 cm) were used to quantify the BBR in cells. A mobile phase consisting of acetonitrile/water (30/70, v/v) containing 0.08% formic acid and 0.15% ammonium acetate was pumped through the column at a speed of 1.0 mL/min. BBR was detected by UV absorbance at a wavelength of 346 nm. Under these conditions, the retention time of BBR was 7.9 min. The quantitative linear range was 12.5–1600.0 ng/mL for BBR. Standard curves of BBR were constructed using weighted linear regression of peak area ratio values of the calibration standards. The correlation coefficient (R^2^) was 0.999.

### Quantification of HIV PIs using HPLC Assay

An Agilent 1200 series HPLC system was used to quantify HIV PIs (AMPV, RITV, and LOPV) concentrations in RAW264.7 macrophages according to the method as previously described with some modifications [31]. Briefly, the process was carried out on a Beckman C18 reverse phase column (5 µm, 4.6 mm×25 cm). The mobile phase, at the flow rate of 1.0 mL/min, consisted of acetonitrile/water (52/48, v/v) containing 0.05 M KH_2_PO4 pH 3.0. The samples were extracted with acetonitrile with a ratio of 40∶60 (v/v) followed by centrifugation. The HIV PIs were detected at a wavelength of 210 nm. Quantification was performed by determining the HPLC peak areas monitored at 210 nm versus the nominal concentration of the analyte. The retention time of AMPV, RITV, and LOPV were 7.1, 14.6 and 17.0 min, respectively. The quantitative linear range was 25.0–5000 ng/mL for AMPV, 100–5000 ng/mL for RITV and LOPV. The correlation coefficient (R^2^) of AMPV, RITV, and LOPV were 0.997, 0.999 and 0.999, respectively.

### Quantification of digoxin using LC/MS Assay

A Shimadzu 2010A liquid chromatograph-mass spectrometer (Shimadzu, Kyoto, Japan) with an ESI source was utilized to quantify intracellular digoxin. Separation was performed using a Inertsil ODS-3 column (150 mm×2.1 mm i.d., 5 µm, GL Science Inc., Japan) fitted with a C18 guard column (4.6×12.5 mm, 5 µm, Agilent, USA) at 40°C. The mobile phase consisting of solvent A (0.02‰ ammonium chloride in water) and solvent B (acetonitrile) using the following gradient: 40–60% B (linear, 0.03 min), 60–80% B (linear, 4.97 min), 80–99% B (linear, 0.03 min), 99% B (1.97 min), 99–40% B (linear, 2.5 min) and 40% B (5.3 min) for equilibration at a speed of 0.2 mL/min. In SIM mode negative ions of digoxin and digitoxin (internal standard) were monitored at m/z 815.20 and 799.30, respectively. Under these conditions, the retention times of digoxin and digitoxin were 6.3 and 4.8 min, respectively. The quantitative linear range was 1.0–50.0 ng/mL for digoxin. The correlation coefficient (R^2^) was 0.999. Digoxin in the cells was extracted using ethyl acetate as described previously [16] and subjected to LC/MS analysis as above.

### Molecular Docking

The structures of ligands including BBR, AMPV, RITV, and LOPV were constructed and optimized under OPLS 2001 force field within Maestro environment. The reported crystal structure of MDR1A/P-gp (PDB code: 3G60) was chosen for the docking template [16]. The protein and ligands were prepared with the Protein Preparation Wizard in Maestro using default options: bond orders were assigned, hydrogens were added, and water molecules were deleted. P-gp was minimized with the OPLS 2001 force field. The ligands were then docked into P-gp flexibly using Glide SP method with default settings.

### RNA isolation and RT-PCR

Total cellular RNA was isolated after treatment using the Promega SV Total RNA Isolation System. The first cDNA was synthesized using the High-Capacity cDNA Archive Kit. RT-PCR was performed as described previously [2]. Primer pairs used were 5′-AGGGCATTTACTTCAAACTTGTC-3′ and 5′-CCTGTCTTGGTCATGTGGTC-3′ for *Abcb1a* (NM_011076), 5′-GTGCTTACTGTCTTCTTCTC-3′ and 5′-CAATGCTTGGCTCG TTATC-3′ for *Abcb1b* (NM_011075), or 5′-GTCGTGGATCTGA CGTGC-3′ and 5′- GATG CCTGCTTCACCAC CTT-3′ for *GAPDH* (BC145810) as an internal control. The PCR products were confirmed by DNA gel electrophoresis and DNA sequencing.

### Western Blot Analysis

The membrane proteins and cytosol proteins were prepared and used for western blot analysis as described previously [32]. The protein concentration was determined using Bio-Rad protein assay reagent. The membrane protein (75 µg) or cytosol protein (25 µg) was resolved on 8% SDS-polyacrylamide gels and transferred to nitrocellulose membranes (BioRad, Hercules, CA, USA). Membranes were blocked with 5% non-fat dry milk in Tris-buffered saline (TBS) for 1 h at room temperature and incubated with the polyclonal antibody to P-gp (C219) (1∶300 dilution, Abcam, Cambridge, MA, USA), or β-actin (1∶500 dilution; Boster Biological Technology, Wuhan, China) at 4°C for overnight. Immunoreactive bands were detected using horse radish peroxidase-conjugated secondary antibody and ChemiDoc XRS^+^ digital imaging system (BioRad, Hercules, CA, USA).

### Statistical analysis

All of experiments were repeated at least three times and the results were expressed as mean ± S.D. One-way ANOVA was employed to analyze the differences between sets of data using GraphPad Prism (GraphPad, San Diego, CA). A value of p<0.05 was considered statistically significant.

## Results

### Effect of HIV PIs on the intracellular accumulation of BBR in macrophages

**Figure 1:**
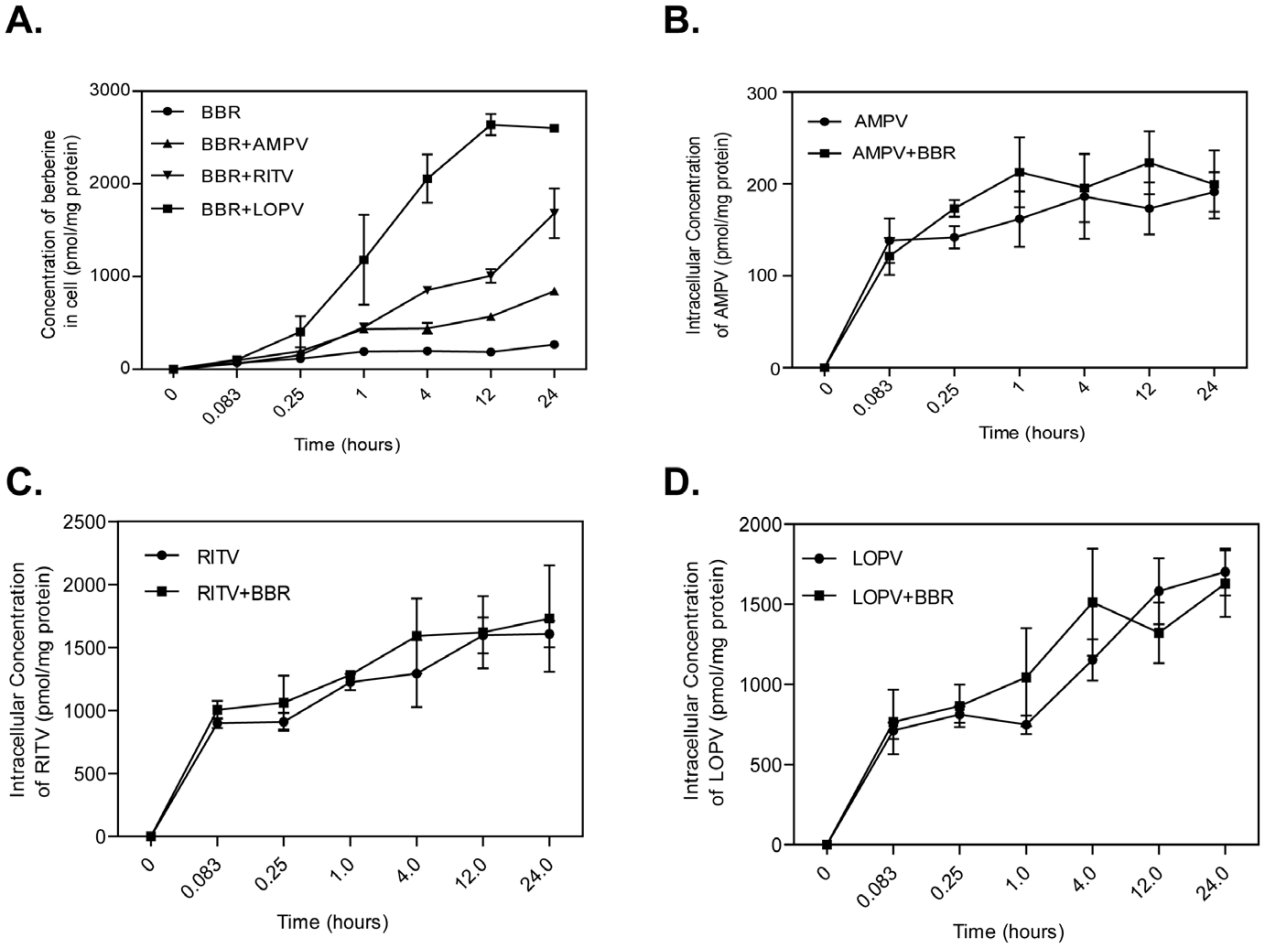
Effect of HIV PIs on BBR uptake in RAW264.7 mouse macrophages. Cells were treated with BBR (5 µM) with or without individual HIV PIs (AMPV, RITV, or LOPV, 15 µM) for 5 min, 15 min, 1 h, 4 h, 12 h or 24 h. The intracellular concentrations of BBR and HIV PIs were determined by HPLC analysis as described in “Methods” and normalized with total protein amount of viable cells. Data are means ± S.D. of three sets of samples. A) Time course of BBR uptake without or with individual HIV PIs; B) Time course of AMPV uptake without or with BBR; C) Time course of RITV uptake without or with BBR; D) Time course of LOPV without or with BBR.

Intracellular BBR uptake studies were performed to evaluate whether HIV PIs (AMPV, RITV and LOPV) affect BBR accumulation in RAW264.7 macrophages. As shown in Fig. 1A, in the absence of HIV PIs, the intracellular concentration of BBR was relatively low. However, in the presence of HIV PIs, the intracellular concentration of BBR was significantly increased. At the 24 h time point, the intracellular BBR concentrations were increased 3-fold, 6-fold and 10-fold, by AMPV, RITV and LOPV, respectively. However, the intracellular concentrations of individual HIV PIs were not affected by BBR in macrophages (Fig. 1B–D).

**Figure 2:**
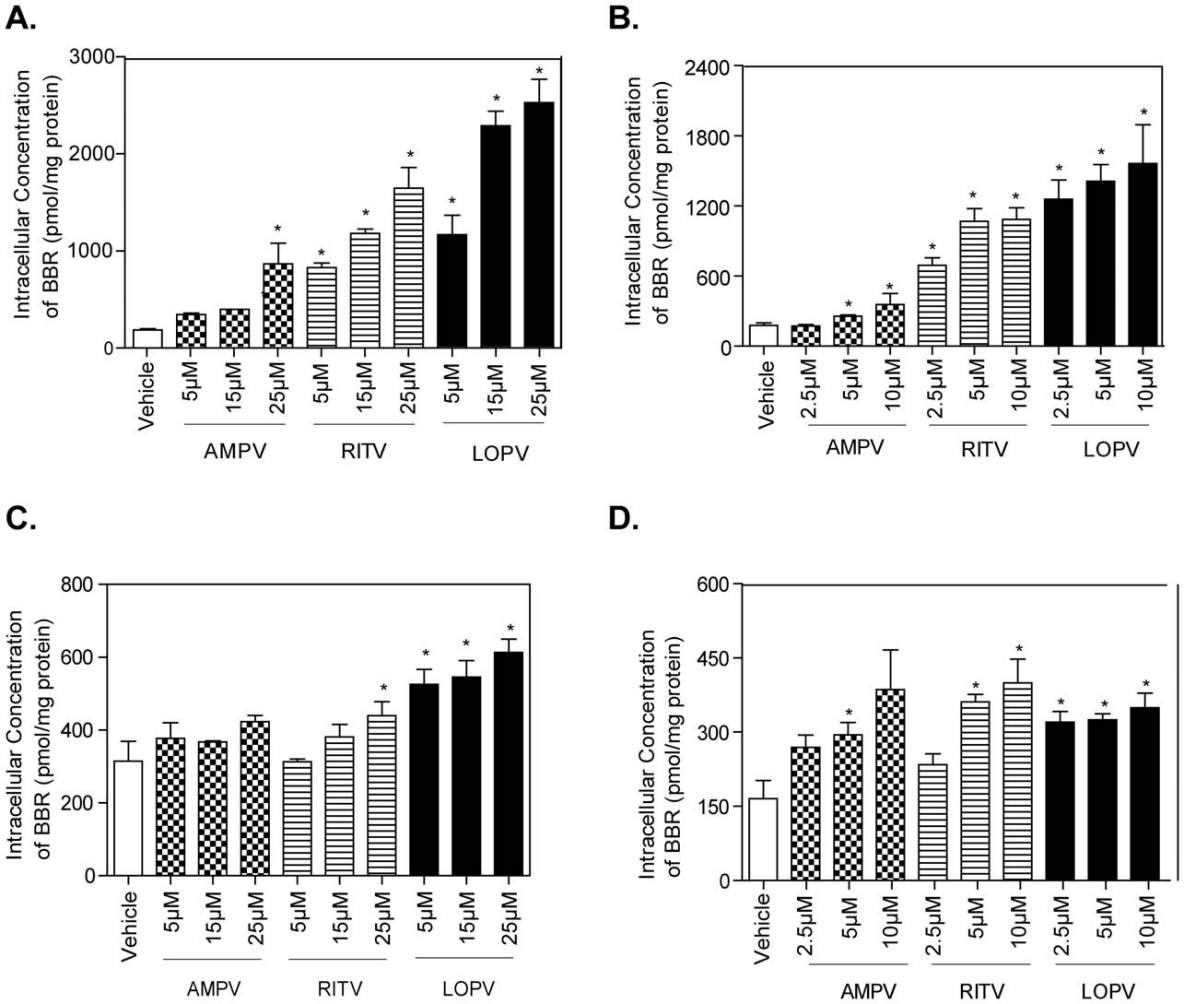
Effect of HIV PIs on BBR uptake in macrophages. A. Intracellular BBR accumulation after exposure to AMPV, RITV, LOPV (5, 15 and 25 µM) for 4 h in RAW 264.7 macrophages. B. Intracellular BBR accumulation after exposure to AMPV, RITV, LOPV (2.5, 5 and 10 µM) for 72 h in RAW 264.7 macrophages. Cells were pretreated with different concentrations of individual HIV PIs for 72 h, then treated with BBR for 4 h. C. Intracellular BBR accumulation after exposure to AMPV, RITV, LOPV (5, 15 and 25 µM) for 4 h in THP-1 macrophages. D. Intracellular BBR accumulation after exposure to AMPV, RITV, LOPV (2.5, 5 and 10 µM) for 72 h in THP-1 macrophages. Cells were pretreated with different concentrations of individual HIV PIs for 72 h, then treated with BBR for 4 h. The intracellular cellular BBR amount was determined by HPLC analysis as described in “Methods” and normalized with total protein amount of viable cells. Data are means ± S.D. of three sets of samples. * *p<0.05*, statistical significance of HIV PI-treated group relative to vehicle control group.

We further examined whether HIV PI-induced increase of BBR accumulation in RAW macrophages was dose-dependent. Cells were treated with BBR in the presence of different concentrations of individual HIV PIs (0, 5, 15, and 25 µM) for 4 h. As shown in Fig. 2A, AMPV, RITV and LOPV, increased BBR accumulation by 5-fold, 9-fold and 14-fold compared to vehicle control at 25 µM, respectively. In parallel with these studies, extended exposure (3 days) of RAW macrophages to HIV PIs (0, 2.5, 5, and 10 µM) also increased intracellular BBR accumulation by 2-fold, 6-fold and 9-fold compared to vehicle control at 10 µM AMPV, RITV or LOPV, respectively (Fig. 2B). Similarly, intracellular BBR concentration in human THP-1 macrophages was significantly increased after acute and extended exposure to HIV PIs (Fig. 2C, D).

### Identification of P-gp as a major player in HIV PI-mediated intracellular accumulation of BBR

**Figure 3:**
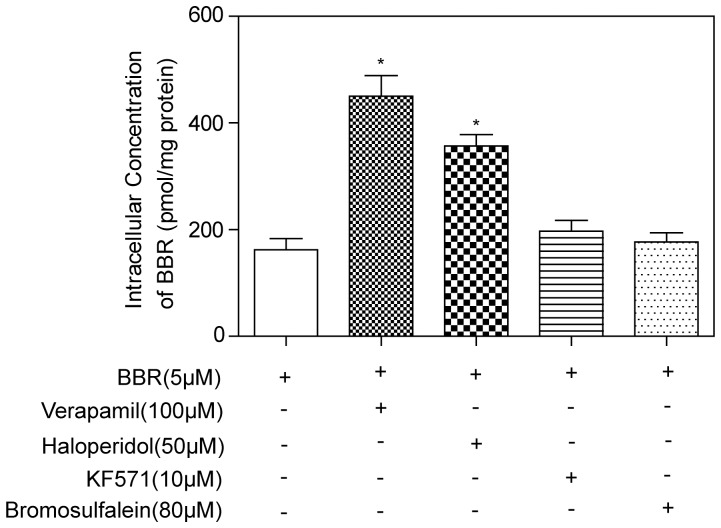
Effect of P-gp and MRP inhibitors on BBR uptake in RAW264.7 macrophages. RAW264.7 macrophages were treated with BBR (5 µM) in the absence or presence of P-gp inhibitors, verapamil (100 µM) and haloperidol (50 µM), or MRP inhibitors, MK571 (10 µM) and bromosulfalein (80 µM) for 4 h. The intracellular concentrations of BBR were determined as described in “Methods”. Data are means ± S.D. of three sets of samples. *p<0.05, statistical significance of transporter inhibitor-treated group relative to BBR group.

To identify the potential drug transporters involved in efflux of BBR in macrophages, the selective chemical inhibitors for P-gp (verapamil and haloperidol) and multidrug resistance-associated protein (MRP) (MK571 and bromosulfalein) were used. As shown in Fig. 3, both verapamil and haloperidol significantly increased uptake of BBR in RAW264.7 macrophages. However, MK571 and bromosulfalein had no effect. The expression of P-gp in RAW264.7 macrophages was confirmed by RT-PCR using specific primers for mouse Mdr1a (Abcb1a) and Mdr1b (Abcb1b). Both Mdr1a and Mdr1b were expressed in RAW264.7 macrophages, but the expression level of Abcb1b was much higher than that of Abcb1a (Online Fig. S1). Taken together, these results indicate that P-gp is the major transporter involved in BBR efflux in macrophages.

**Figure 4:**
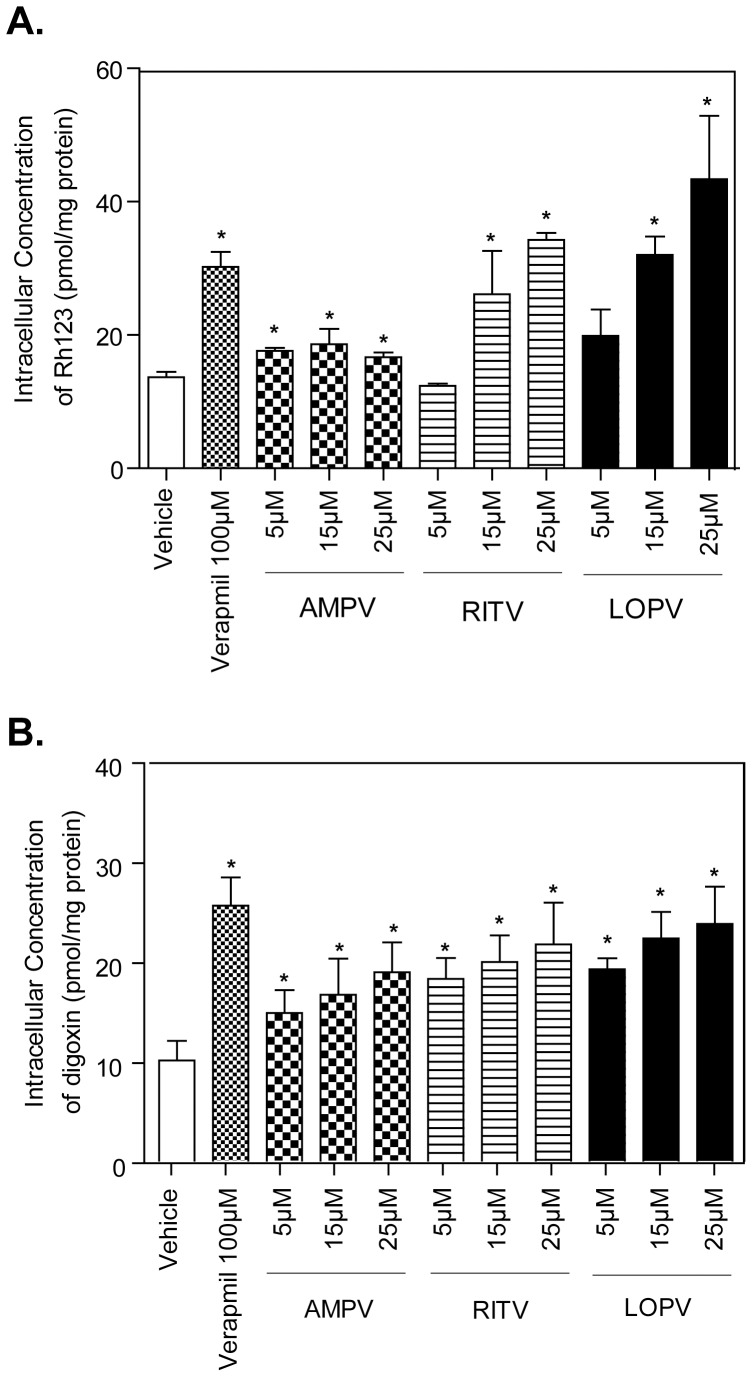
Effect of HIV PIs on P-gp transporter activity in RAW264.7 macrophages. A. Intracellular Rh123 accumulation after exposure to AMPV, RITV, LOPV (5, 15 and 25 µM), and verapamil (100 µM) for 4 h. B. Intracellular digoxin accumulation after exposure to AMPV, RITV, LOPV (5, 15 and 25 µM), and verapamil (100 µM) for 4 h. Data are means ± S.D. of three sets of samples. * *p<0.05*, statistical significance of HIV PI-treated group relative to vehicle control group.

We further examined the effect of HIV PIs on P-gp transporter expression and activity in macrophages. The P-gp protein expression levels were determined by Western blot analysis. The results indicated that HIV PIs and BBR had no effect on P-gp protein expression (Online Fig. S2). Mouse macrophages were treated with different concentrations of individual HIV PIs (0, 5, 15, and 25 µM) in the presence of Rh123 or digoxin for 4 h. The intracellular concentrations of Rh123 and digoxin were determined as described in the Methods section. As shown in Fig. 4A and B, HIV PIs dose-dependently increased the intracellular Rh123 and digoxin amounts, indicating HIV PIs inhibited P-gp activity. These results indicated that HIV PIs inhibited P-gp activities to different extents, and the greatest inhibition was observed with LOPV treatment, followed by RITV and AMPV.

**Figure 5:**
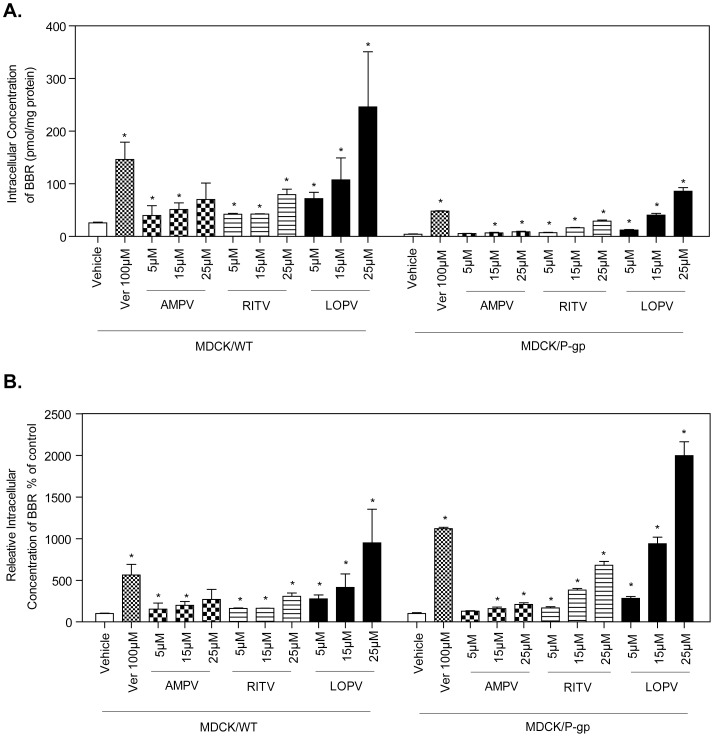
Influence of HIV PIs on BBR uptake in MDCK cells. Wild-type MDCK and P-gp-transfected MDCK cells were treated with BBR (5 µM) in the presence of different amount of individual HIV PIs (5, 15, and 25 µM) or verapamil (100 µM) for 4 h, respectively. The intracellular BBR amount was measured by HPLC analysis as described in “Methods” and normalized with total protein amount of viable cells. Data are means ± S.D. of three sets of samples. * p<0.05 statistical significance of HIV PI-treated group relative to BBR group. **A.** Intracellular BBR concentration after exposure to AMPV, RITV, LOPV, and verapamil for 4 h in MDCK/WT and MDCK/P-gp cells. **B.** Relative changes of intracellular BBR concentration compared to basal levels after treatment with HIV PIs and verapamil for 4 h in MDCK/WT and MDCK/P-gp cells.

In order to further define the role of P-gp in HIV PI-induced intracellular accumulation of BBR, the wild type MDCK (MDCK/WT) and human P-gp-transfected MDCK (MDCK/P-gp) cell lines were used. The P-gp expression level in MDCK/WT is minimal compared to that in MDCK/P-gp cells. As expected, the intracellular concentration of BBR was significantly increased both in wild type and P-gp-transfected MDCK cells in the presence of increasing concentrations of individual HIV PIs (Fig. 5A). However, the BBR concentrations in MDCK/P-gp cells treated with HIV PIs and verapamil were still lower than those in wild type MDCK cells, indicating the incomplete inhibition of over-expressed P-gp by HIV PIs and verapamil in MDCK/P-gp cells. As shown in Fig. 5B, the percentage of increase of intracellular BBR concentration induced by verapamil and HIV PIs (RITV and LOPV) in MDCK/P-gp cells was much higher than that in MDCK wild type cells. These results do suggest that inhibition of P-gp activity may represent the major mechanism underlying HIV PI-induced increase of intracellular accumulation of BBR in macrophages.

### Molecular Docking of BBR and HIV PIs to P-gp

**Figure 6:**
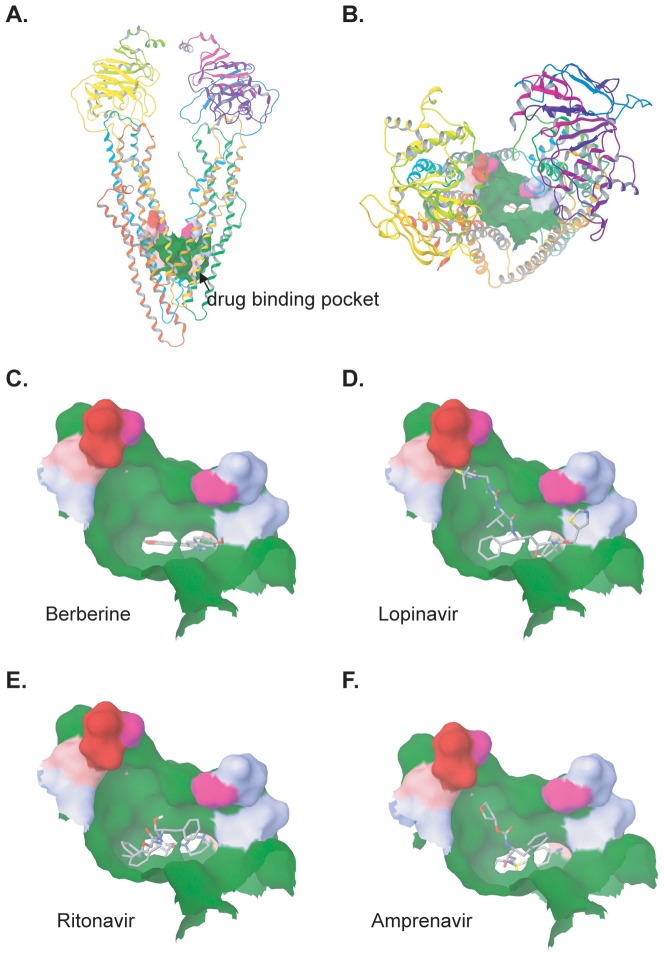
Molecular docking of BBR and HIV PIs to mouse P-gp. A and B: side and top view of the drug binding pocket of P-gp, respectively. Most of the surface of binding pocket colored green is hydrophobic and aromatic residues. C–F: The overall docking views of BBR, AMPV, RITV, and LOPV in the binding pocket.

Recently, the crystal structure of mouse MDR1a/P-gp (ABCB1) was identified, which has 87% sequence identity to human P-gp, in a drug-binding-competent state [16]. We therefore utilized the mouse P-gp structure as a receptor to perform molecular docking to test BBR and HIV PIs. The results suggest that BBR and HIV PIs were capable of binding to the drug binding pocket, but at different binding locations (Fig. 6). The binding site of BBR was located in the “lower” part of binding pocket (Fig. 6C); while the docked structure of LOPV occupied the whole “upper” part of the binding pocket (Fig. 6D). In addition, the binding sites of RITV and AMPV (Fig. 6E and F) were located at the “middle” part of the pocket. This suggests that the binding of HIV PIs in the drug binding pocket could competitively inhibit BBR from binding to the deeper site of this internal cavity. But binding of BBR has no effect on the binding of HIV PIs to their binding sites. In addition, LOPV, RITV and AMPV docked with higher glide energy to the binding pocket, compared to BBR. The ranking order of glide energy is: LOPV(−61.1 kcal/mol) >RITV(−56.6 kcal/mol) >AMPV (−49.5 kcal/mol) >BBR (−33.6 kcal/mol). These data suggest that HIV PIs strongly bind to P-gp and prevent P-gp-mediated efflux of BBR in macrophages.

## Discussion

HIV PIs are core components of HAART for HIV infection. CAMs have been extensively studied for use in HIV patients to manage HAART-associated side effects and improve overall physical health [33], [34]. However, both HIV PIs and many CAMs are reported to interact with drug transporters and metabolizing enzymes [35], [36]. As such, CAMs could potentially affect the beneficial outcome of HAART or the components of HAART could complicate the beneficial effects of CAMs. Limited studies have been published related to interactions between CAMs and anti-HIV drug regimens. Macrophages are the major target of HIV infection and also play critical roles in inflammation and cardiovascular diseases. We have previously shown that BBR prevents HIV PI-induced inflammatory response through modulating ER stress signaling pathways in macrophages and the intracellular concentration of BBR is significantly increased by HIV PIs (Fig. 1). However, the mechanism of HIV PI-induced BBR accumulation within macrophages is not previously known and was the focus of the current study.

Most of the recent studies regarding the role of BBR and HIV PIs in regulating drug transporters were done in intestinal epithelial cells, hepatocytes, or cancer cell lines [17], [18], [23], [37], [38], [39]. Consistent with previous studies in other cell types, the accumulation of BBR was shown to be energy dependent in macrophages [18], [39], suggesting ABC-transporters may be involved. It has been shown that P-gp mediates efflux of BBR in Caco-2 cells [17]. However, little is known regarding the expression and function of P-gp in macrophages. RT-PCR analysis indicated that P-gp is highly expressed in RAW264.7 macrophages (online Fig. S1). Previous studies in the literature is contradictory regarding the role of HIV PIs in the modulation of drug transporters [22], [23], [25], [40], [41]. Some of studies state that HIV PIs have a direct inhibitory effect on the activity of P-gp [22], [40], whereas others report an increase in P-gp activity with treatment of HIV PIs [23], [25], [41]. Such discrepancies may be a result of varying concentrations and duration of treatment with different HIV PIs in different types of cells. In our studies, we did not observe significant changes of P-gp expression after HIV PIs and BBR treatment (online Fig. S2). By using selective substrates and inhibitors, we were able to determine the effect of HIV PIs on P-gp activities in macrophages. Transport of the prototypical P-gp substrates Rh123 and digoxin in cell culture has been successfully used to assess P-gp activity [42]. Rh123 and digoxin cell exclusion studies in RAW264.7 macrophages showed that intracellular concentrations of digoxin and Rh123 were significantly increased in macrophages after exposure to various HIV PIs. In RAW264.7 macrophages the ranking order of inhibition of P-gp activity was LOPV>RITV>AMPV. BBR accumulation was significantly increased in macrophages after acute and extended exposure to various HIV PIs. These findings suggest that the inhibitory effect of HIV PIs on P-gp activity was unidirectional, unlike atazanavir, which inhibits P-gp activity in short-term treatment and induces P-gp activity in long-term treatment [37]. Similar to the findings in mouse macrophages, HIV PIs also increased intracellular concentration of BBR in human THP-1 macrophages wild type MDCK cells and P-gp-transfected MDCK cells, with a rank order of patency LOPV>RITV>AMPV. Moreover, a lower multiple of the increase in BBR concentration after individual HIV PIs or Verapamil treatment in MDCK cells was observed in wild type MDCK cells (Fig. 5B), which may due to the lower levels of endogenous P-gp expression. The P-gp expression level in wild type MDCK cells is about 4% of that in P-gp-transfected MDCK cells [43].

The molecular docking studies further suggest that the inhibitory effect of individual HIV PIs on the P-gp transporter is as follows: LOPV>RITV>AMPV. These results also suggest that HIV PIs could competitively block the binding of BBR to its binding site in P-gp, while BBR has no reverse effect on the binding of HIV PIVs to their binding sites in P-gp. Taken together, our studies suggest that HIV PIs increase BBR concentrations mainly by inhibiting the activities of P-gp. It should be noted that it has recently been reported that HIV PIs are also inhibitors of breast cancer resistance protein (BCRP) and multidrug resistance-associated protein1 (MRP1) [44], [45], [46]. However, the expression of BCRP in murine macrophages has not been clearly identified and the role of MRPs and BCRP in the accumulation of BBR increased by HIV PIs remains to be established in our future study.

It has been long realized that the bioavailability of BBR is very low *in vivo* [47]. Several possible mechanisms have been identified for its poor bioavailability [17], [48]. P-gp-mediated efflux represents a major mechanism. Although inhibition of efflux of BBR by coadministration of HIV PIs may intuitively cause concern for use in clinic, this specific drug interaction may actually be beneficial to improve the biological activities of BBR. We will examine the effect of HIV PIs on bioavailability of BBR using an *in vivo* mouse model and further define the interaction between BBR and HIV PIs with other transporters in our future study.

In summary, drug interactions of BBR with HIV PIs mediated by P-gp inhibition were suggested by *in vitro* studies using macrophages. Although further *in vivo* investigations of possible interactions are necessary, the current study provided valuable information for understanding the underlying cellular mechanism of BBR-HIV PIs interactions, which is critical to effectively applying this combinational therapy in the clinic.

## Supporting Information

Figure S1 **Expression of P-gp in macrophages.** Total cellular RNA was isolated from RAW264.7 macrophages and reverse transcribed into 1^st^ cDNA. Specific primers for MDR1a/P-gp (ABCB1a) and MDR1b/P-gp (ABCB1b) were used to run PCR. The PCR products were analyzed by DNA electrophoresis and confirmed by DNA sequencing. Representative image is shown.
(PDF)

Figure S2 **Effect of HIV PIs and BBR on P-gp expression in RAW264.7 macrophages.** Representative immunoblots against P-gp and β-actin from the membrane and cytosol extracts of RAW macrophages treated with individual HIV PIs (15 µM) and BBR (5 µM) for 6 h are shown. Blot shows specific bands of P-gp at ∼170 kDa and β-actin was used as loading control.
(PDF)
