# Supplementary figures and images for: Inhibition of P-Glycoprotein by HIV Protease Inhibitors Increases Intracellular Accumulation of Berberine in Murine and Human Macrophages
Source: PLoS One. 2013 Jan 23;8(1):e54349. doi: 10.1371/journal.pone.0054349 (PMC3553168; doi:10.1371/journal.pone.0054349)

**Figure S1**

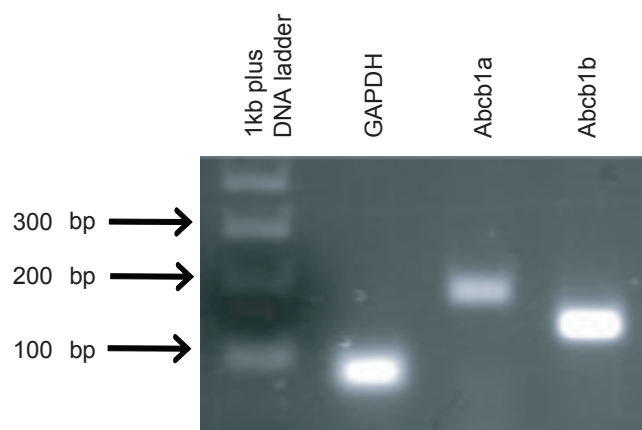

Supplement: Figure S1 — Expression of P-gp in macrophages. Total cellular RNA was isolated from RAW264.7 macrophages and reverse transcribed into 1st cDNA. Specific primers for MDR1a/P-gp (ABCB1a) and MDR1b/P-gp (ABCB1b) were used to run PCR. The PCR products were analyzed by DNA electrophoresis and confirmed by DNA sequencing. Representative image is shown. (PDF) [file pone.0054349.s001.pdf]

**Figure S2**

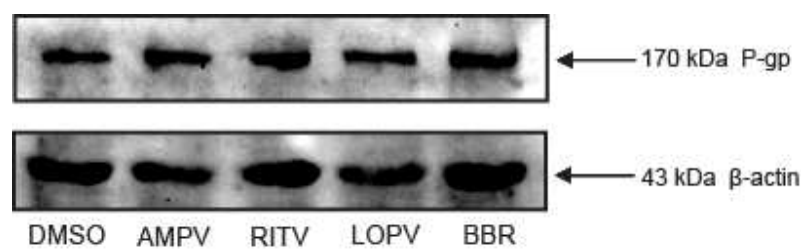

Supplement: Figure S2 — Effect of HIV PIs and BBR on P-gp expression in RAW264.7 macrophages. Representative immunoblots against P-gp and β-actin from the membrane and cytosol extracts of RAW macrophages treated with individual HIV PIs (15 µM) and BBR (5 µM) for 6 h are shown. Blot shows specific bands of P-gp at ∼170 kDa and β-actin was used as loading control. (PDF) [file pone.0054349.s002.pdf]
